# Quantitative analysis of the direct piezoelectric response of bismuth ferrite films by scanning probe microscopy

**DOI:** 10.1038/s41598-019-56261-w

**Published:** 2019-12-23

**Authors:** Kento Kariya, Takeshi Yoshimura, Katsuya Ujimoto, Norifumi Fujimura

**Affiliations:** Department of Physics and Electronics, Graduate School of Engineering, Osaka Prefecture University, Sakai, 599-8531 Japan

**Keywords:** Ferroelectrics and multiferroics, Scanning probe microscopy

## Abstract

Polarisation domain structure is a microstructure specific to ferroelectrics and plays a role in their various fascinating characteristics. The piezoelectric properties of ferroelectrics are influenced by the domain wall contribution. This study provides a direct observation of the contribution of domain walls to the direct piezoelectric response of bismuth ferrite (BiFeO_3_) films, which have been widely studied as lead-free piezoelectrics. To achieve this purpose, we developed a scanning probe microscopy-based measurement technique, termed direct piezoelectric response microscopy (DPRM), to observe the domain structure of BiFeO_3_ films via the direct piezoelectric response. Quantitative analysis of the direct piezoelectric response obtained by DPRM, detailed analysis of the domain structure by conventional piezoelectric force microscopy, and microscopic characterisation of the direct piezoelectric properties of BiFeO_3_ films with different domain structures revealed that their direct piezoelectric response is enhanced by the walls between the domains of spontaneous polarisation in the same out-of-plane direction.

## Introduction

Piezoelectric films have attracted much attention as sensing and actuating components in microelectromechanical systems (MEMSs)^1,2^. In addition to practical applications such as inkjet printer heads and gyro-sensors, various piezoelectric MEMS devices, including microphones, speakers, optical microscanners, ultrasonic transducers, and energy harvesters, have been actively studied for future practical use. Although lead zirconate titanate [Pb(Zr,Ti)O_3_, PZT] and aluminium nitride films are already widely used in MEMS devices, there is high demand to further improve piezoelectric properties to enhance device performance. Specifically, lead-free piezoelectrics are currently in demand from the viewpoint of environmental protection.

We developed piezoelectric MEMS vibration energy harvesters using PZT and bismuth ferrite (BiFeO_3_) films^3–5^. The *e*_31,*f*_ of BiFeO_3_ films is approximately −3.5 C/m^2^, which is lower than that of PZT films. Nonetheless, BiFeO_3_ films have a sufficient figure of merit (FOM) for energy harvesting applications, in which the FOM is given by ${e}_{31,f}^{2}/{\varepsilon }_{0}{\varepsilon }_{r}$ (where ε_0_ and ε_r_ are the permittivity of vacuum and the relative permittivity, respectively)^6,7^ because of their low permittivity (~100)^8,9^. In fact, we have demonstrated that harvesters using BiFeO_3_ films show output power comparable to that obtained using PZT films^10^. Similarly, it can be expected that BiFeO_3_ films will be suitable for sensing applications because of their large piezoelectric voltage constant $(\,\propto {e}_{31,f}/{\varepsilon }_{r})$. From the viewpoint of the application of BiFeO_3_ films, the direct piezoelectric response is more important than the converse piezoelectric response; however, most studies on BiFeO_3_ films have only characterised the latter^11–15^. We have investigated the relationship between the *e*_31,*f*_ coefficient (determined by the direct piezoelectric response) and the crystal and domain structures using epitaxial and oriented BiFeO_3_ films^16–20^. It was found that films with a higher domain wall density have a larger direct piezoelectric response. For further improvement of the *e*_31,*f*_ coefficient of BiFeO_3_ films, an understanding of the mechanisms is important.

Recently, Tsujiura *et al*.^21^ reported that the effective transverse piezoelectric stress coefficient (*e*_31,*f*_) values of PZT films determined via direct and converse piezoelectric effects do not coincide. This discrepancy was not observed for polar wurtzite films^22^. These results suggest that the domain wall contribution to the piezoelectric properties of PZT films is not negligible. Although the domain wall contribution to the converse piezoelectric response has been investigated in various ways, its contribution to the direct piezoelectric response has not. As mentioned above, the direct piezoelectric response is important for sensing and energy harvesting applications. However, there is no method to characterize it on the nanoscale.

In this article, we observe the ferroelectric domain structure of epitaxially grown BiFeO_3_ films and quantitatively measure the longitudinal piezoelectric stress coefficient (e_*33*,*f*_) at high spatial resolution through direct piezoelectric response microscopy (DPRM), which was developed in this study. In DPRM, a modulated mechanical force is applied to the nano-region of the film surface by a conductive AFM probe. The current induced by the direct piezoelectric response is detected by a current-to-voltage (*I*/*V*) converter, which is a major difference from previously reported piezoelectric force microscopy (PFM), atomic force acoustic microscopy (AFAM)^23^, ultrasonic force microscopy (UFM)^24,25^ and other methods. The details of DPRM and comparison with conventional PFM are described in the Methods section. Although Gomez and co-workers measured the direct piezoelectric response using scanning probe microscopy (SPM), the spatial resolution and sensitivity are insufficient for this purpose^26^. By combining the DPRM results with analysis by conventional PFM, we can discuss the enhancement of the direct piezoelectric response of BiFeO_3_ films at domain walls.

## Results and Discussion

**Figure 1:**
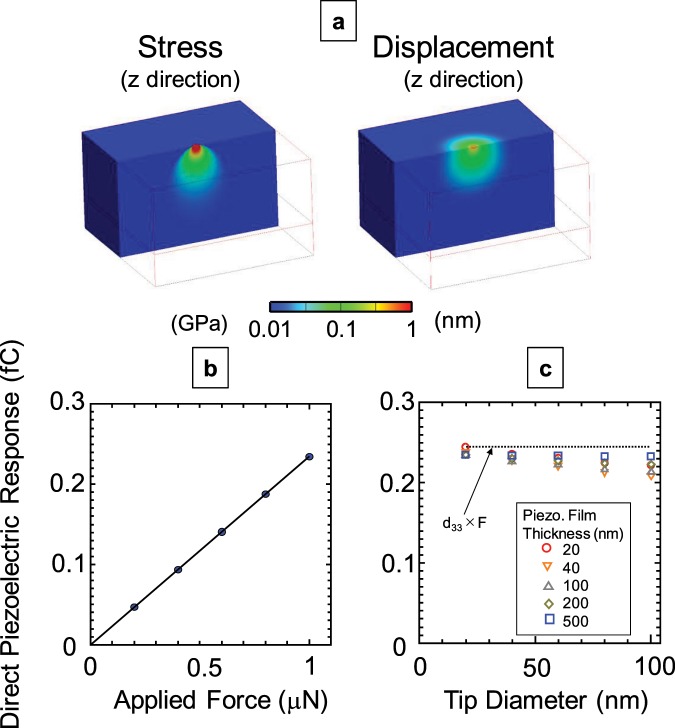
Finite element analysis for DPRM. (**a**) Stress (left) and surface deformation (right) along the z direction. Dependence of the direct piezoelectric response via the cantilever on the (**b**) applied mechanical force and (**c**) thickness of the piezoelectric film and the diameter of the SPM tip.

The output signal of DPRM was analysed by the finite element method (Femtet, Murata Software Co., Ltd., Tokyo, Japan). In the simulations, a force (*F*) was applied from the AFM tip to the piezoelectric film. Figure 1(a) shows the stress and surface deformation in the out-of-plane direction. The PZT film is assumed in this calculation because the matrixes of the elastic compliance, piezoelectric constant and dielectric permittivity are available. The analysis was carried out with an *F* of 1 μN; tip diameter (*D*) of 20 nm; and thickness (*h*), Young’s modulus (*E*), and *d*_33_ of the piezoelectric film of 200 nm, 73 GPa, and 245 pm/V, respectively. The stress was distributed in the film, and the surface around the tip was deformed. Figure 1(b) displays the relationship between the induced charge (*Q*) via the direct piezoelectric response and *F* under the same conditions as in Fig. 1(a). We found that *Q* increased linearly with *F* and that the proportional relationship was simply *Q* = *d*_33_*F* despite the application of uneven stress. Moreover, this relationship was almost unaffected under other conditions, as illustrated in Fig. 1(c). In this analysis, *D* and *h* were varied from 20 to 100 nm and from 20 to 500 nm, respectively. The charges were almost independent of *D* and *h*, which suggests that DPRM can achieve quantitative analysis regardless of the measurement conditions and sample structure. Moreover, the direct piezoelectric response was hardly influenced by other contributions, such as the electrorestrictive and charging effects, because no electric field was applied in the measurement^27^. Therefore, DPRM should have the ability to measure the pure piezoelectric response of ultrathin piezoelectric films, which is an advantage over conventional PFM.

The samples used were (100) and (111) BiFeO_3_ epitaxial thin films grown on (100) SrRuO_3_/(100) SrTiO_3_ and (111) SrRuO_3_/(111) SrTiO_3_ substrates, respectively, using pulsed laser deposition. The details of the samples are described elsewhere^15^. The thickness of these films was 500 nm. X-ray reciprocal space mapping indicated that both films had rhombohedral structures. The macroscopic remnant polarisation of the (111) and (100) films was 95 and 56 μC/cm^2^, respectively, and their *e*_31,*f*_ value was −1.3 and −3.1 C/m^2^, respectively^15^.

**Figure 2:**
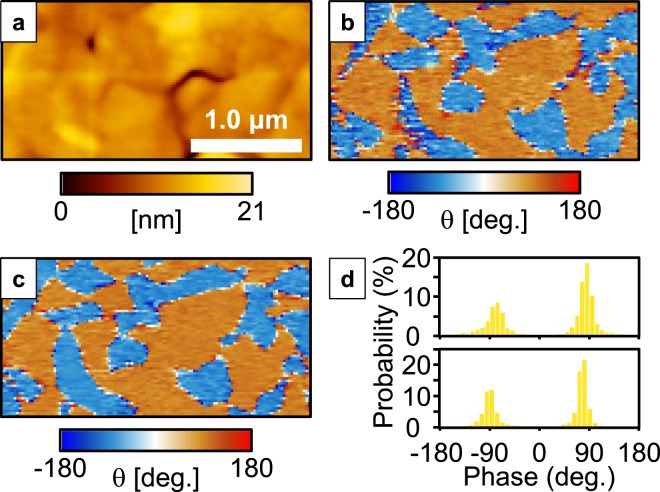
DPRM measurement of a (100) BiFeO_3_ epitaxial film. (**a**) Surface morphology of the film. Phase images obtained by (**b**) DPRM and (**c**) PFM. (**d**) Frequency spectrum of the phase image obtained by DPRM (top) and PFM (bottom).

Figure 2(a) shows a topographic image obtained by DPRM for the (100) BiFeO_3_ film. A compressive force modulation of 1300 nN was applied. The topographic DPRM image was almost identical to that of conventional SPM, which indicates that the sinusoidally applied force did not influence the topology observation and that constant contact of the AFM probe with the sample was maintained. Figure 2(b) depicts the corresponding phase image of DPRM, which maps the phase difference between the applied force and the direct piezoelectric response. The phase signals (*θ*) of 90° and −90° correspond to the upward and downward domains, respectively. The domain pattern was clearly observed by DPRM. The obtained pattern is almost identical to the PFM phase image shown in Fig. 2(c). The histograms of the phase signals for DPRM and PFM in Fig. 2(d) indicate that DPRM has almost the same spatial resolution as PFM. The phase difference of 180° between the upward and downward domains indicates that the signal originates primarily from the direct piezoelectric response. In contrast to this result, Lu *et al*.^28^ reported that polarisation switching was induced by the application of the strain gradient generated by the SPM tip. Although the measurement method of Lu *et al*. was similar to that of DPRM, such phenomena were not observed herein, as shown in Fig. S1, in which direct piezoelectric response mapping images were obtained under force modulation in the range of 600–2300 μN. It seems that the difference between their method and DPRM is the electrical condition of the AFM probe. In DPRM, no electric field is applied to the film because an imaginary short circuit is formed by the *I*/*V* converter.

**Figure 3:**
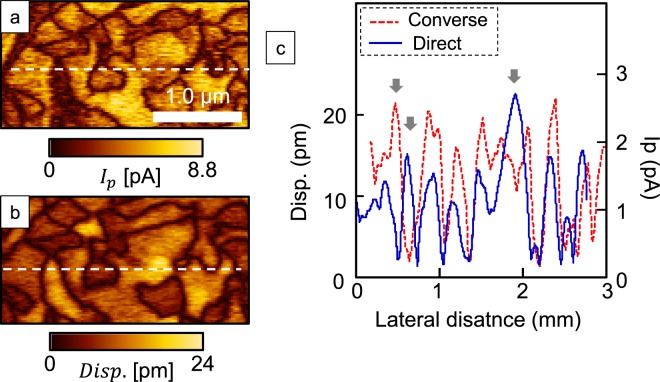
Comparison of mapping images obtained by DPRM and PFM. Absolute (**a**) DPRM and (**b**) PFM mapping results. (**c**) Cross-sectional images on the lines depicted in (**a**,**b**), respectively.

Figure 3(a,b) show the DPRM and PFM amplitude images, respectively. These images indicate that the signal-to-noise ratio of DPRM was comparable to that of PFM. While the domain patterns are almost the same in these two images, the amplitude distributions differ. The cross sections of these piezoelectric responses at the white dashed line are shown in Fig. 3(c). In the domains indicated by grey arrows, the domains with a large direct piezoelectric response have a small converse piezoelectric response and vice versa. Thus, the direct and converse piezoelectric responses have different active regions. This result is consistent with a previous comparison of direct and converse piezoresponses in our BiFeO_3_ films. The relationship between *e*_31,*f*_ (determined from the direct piezoelectric response) and the effective longitudinal piezoelectric coefficient, *d*_33(AFM)_, determined from the converse piezoelectric response using SPM, is summarised in Fig. S2. A strong correlation was not observed.

**Figure 4:**
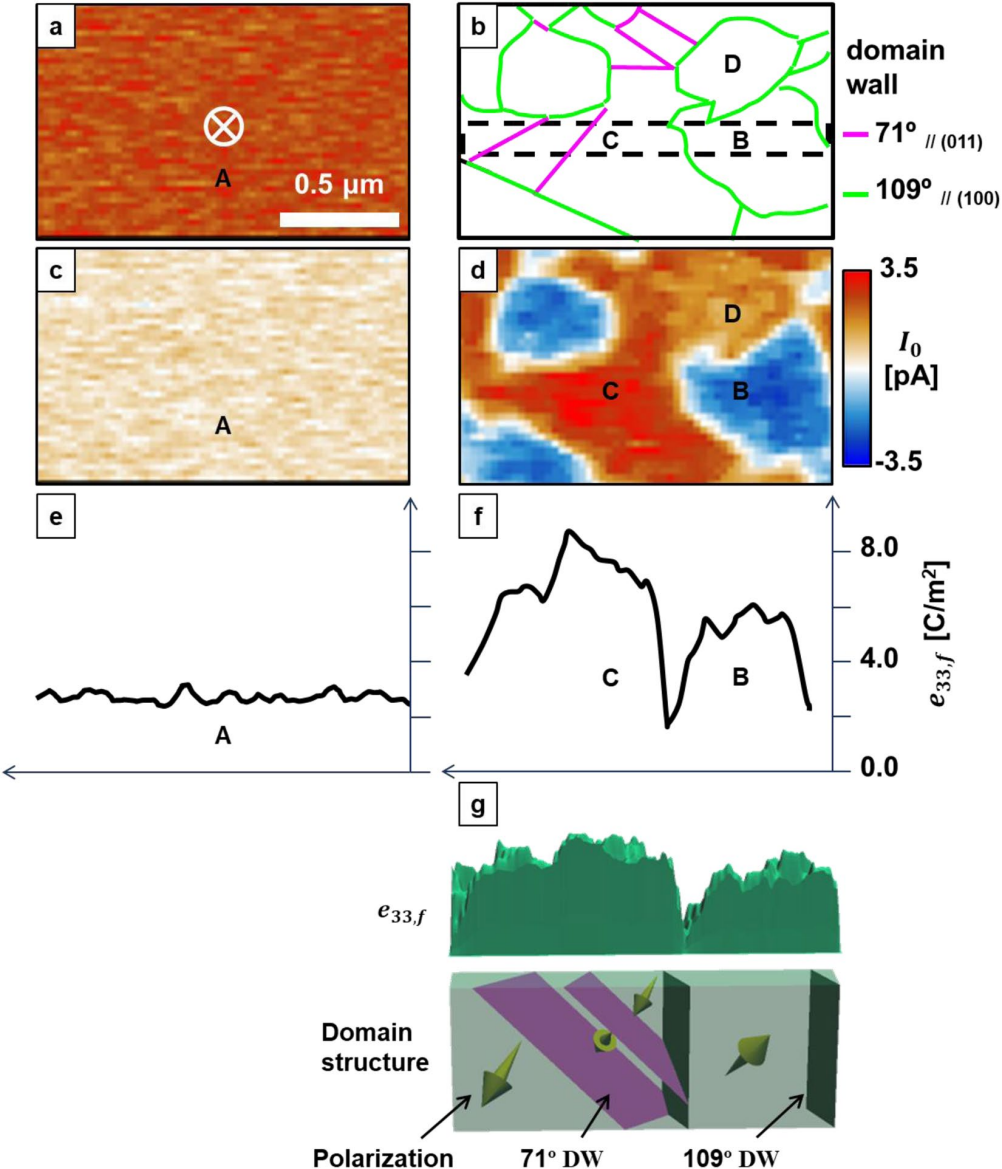
Direct piezoelectric response of (111) and (100) BiFeO_3_ epitaxial films (left and right, respectively). (**a**) Vertical PFM. (**b**) Domain structure determined by PFM. (**c**), (**d**) DPRM images. (**e**,**f**) Average e_33,f_ cross-section of the areas inside the dashed black rectangles. (**g**) Schematic of the relationship between e_33,f_ determined by DPRM and the domain structure. DW indicates the domain wall.

The contribution of the domain walls to the direct piezoelectric response of the BiFeO_3_ films was investigated by combining the PFM and DPRM measurements. Figure 4(a) shows the domain structure of the (111) film measured by PFM. The (111) film had a single domain structure with downward spontaneous polarisation; domain walls were not observed. Figure 4(b) shows the domain structure of the (100) film determined from the vertical and lateral PFM images. The details of the determination are provided in Fig. S3. The (100) film has a multidomain structure composed of 71° and 109° domain walls. The DPRM images of the *I*_*p*_sin*θ* signal of the (111) and (100) films are presented in Fig. 4(c,d), respectively. The (111) film shows a homogeneous signal distribution, which is consistent with the results shown in Fig. 4(a) and the quantitative analysis by DPRM discussed above. In contrast, the (100) film exhibits a broad distribution of the amplitude of *I*_*p*_. To investigate the origin of this distribution in detail, *e*_33,*f*_ was calculated using

1 $${e}_{33,f}={d}_{33}E=\frac{E{I}_{p}}{2\pi fkx}$$

where *k* is the spring constant of the AFM probe and *f* and *x* are the operating frequency and displacement of the actuator, respectively. The BiFeO_3_ films were assumed to have *E* = 170 GPa. The cross sections of *e*_33,*f*_ distribution for the (111) and (100) films are shown in Fig. 4(e,f), respectively. The area inside the dashed black rectangles in Fig. 4(b) was used for this calculation. From Fig. 4(e), the average *e*_33,*f*_ is calculated to be 2.7 C/m^2^, which corresponds to the intrinsic piezoelectric response of the (111) film because this film has a single domain structure (denoted domain A). In contrast, the (100) film has different *e*_33,*f*_ values depending on the domain, as shown in Fig. 4(f). The domain surrounded by 109° domain walls (domain B) has an average *e*_33,*f*_ of 5.7 C/m^2^. The domain with 71° domain walls (domain C) has an average *e*_33,*f*_ of 7.3 C/m^2^, which is 30% larger than that of domain B. The difference between the *e*_33,*f*_ values of domains A and B must originate from the domains’ engineered configuration because all of the BiFeO_3_ films used in this study have rhombohedral structures. The increase in *e*_33,*f*_ in domain C of 1.6 C/m^2^ compared with that in domain B indicates the contribution of the 71° domain walls to the direct piezoelectric response. This enhancement is not caused by the difference in the spontaneous polarisation directions because domain D, which has the same spontaneous polarisation direction as that of domain C and no 71° domain walls, has a smaller *e*_33,*f*_ than domain C. Moreover, the 71° domain walls are tilted with respect to the film surface. It appears that this tilt contributed to the increase in *e*_33,*f*_ in the peripheral area of the 71° domain walls. The maximum *e*_33,*f*_ was observed in the region with two 71° domain walls, as shown in Fig. 4(g). This is also consistent with the domain wall contribution to the piezoelectric response of domain-engineered ferroelectrics reported by Wada and colleagues^29^. The enhancement of the direct piezoelectric response at the 71° domain walls was observed with good reproducibility in regions other than domain C, as shown in Fig. S4, and can be explained by the broadening of the domain walls induced by a stimulus, as proposed by Rao and Wang^30^.

Finally, the contribution of the 71° domain walls to the macroscopic direct piezoelectric response characterised by *e*_31,*f*_ is discussed. For the (111) film, which has only an intrinsic contribution, the ratio of *e*_31,*f*_ (=−1.3 C/m^2^) to *e*_33,*f*_ (=2.7 C/m^2^) is −2.1. As $|{d}_{31}|/{d}_{33}$ of perovskite ferroelectrics is approximately 0.4^31^, the obtained ratio is reasonable, which suggests quantitative analysis with DPRM. By applying this ratio to *e*_33,*f*_ of domain B (5.7 C/m^2^), the estimated intrinsic contribution of *e*_31,*f*_ in the (100) film is −2.7 C/m^2^. The difference between the measured *e*_31,*f*_ (=−3.5 C/m^2^), and the intrinsic contribution of *e*_31,*f*_ is −0.8 C/m^2^, which corresponds to the contribution of the 71° domain walls. This value is consistent with that estimated from the enhancement of *e*_33,*f*_ caused by the contribution of the 71° domain walls, which is 1.6 C/m^2^. Because the 71° domain walls are mainly formed between the domains of spontaneous polarisation in the same out-of-plane direction, these walls will remain after the poling treatment and contribute to the enhancement of the direct piezoelectric response. The domain wall contribution to the direct piezoelectric response of the BiFeO_3_ films is only 30% of the intrinsic contribution. This is important for energy conversion applications such as energy harvesting because the electromechanical coupling factor (*k*^2^) is proportional to the square of the piezoelectric coefficient. It is estimated that almost half of *k*^2^ originates from the domain wall contribution.

## Conclusions

This work presented a technique to observe the domain structure of ferroelectric films through the direct piezoelectric effect and quantitatively characterise the effective longitudinal piezoelectric coefficient. Using the developed DPRM technique, a marked increase in the direct piezoelectric response in the domains with 71° domain walls was identified. This work should stimulate attempts to enhance the piezoelectric properties of ferroelectric films through the modification of domain structures, including the introduction of domain walls.

## Methods

**Figure 5:**
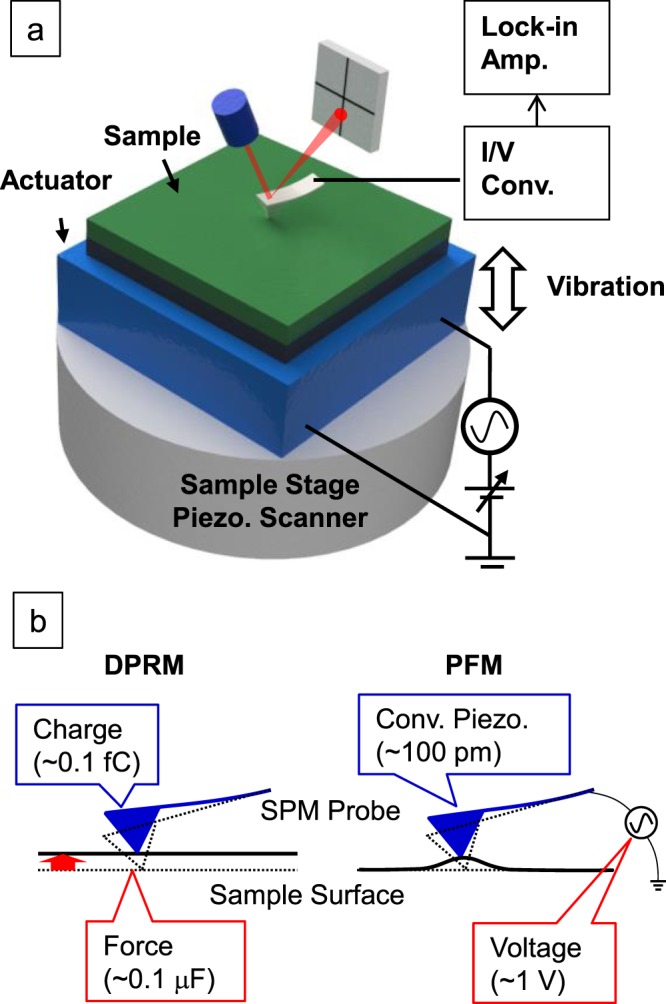
(**a**) Schematic illustration of the experimental setup for DPRM. (**b**) A comparison between DPRM and conventional PFM.

An SPM (SII, NanoNAvi) was modified to observe the direct piezoelectric response of a nanoscopic region. A schematic illustration of DPRM is shown in Fig. 5(a). To apply a modulated mechanical force to a sample, a piezoelectric actuator was placed on the sample stage of the SPM. The sample was set on the actuator. A conductive AFM probe was put in contact with the sample surface, and then the actuator was operated at a higher frequency than the cutoff frequency of the low-pass filter within the feedback controller of SPM, which avoided cancellation of the applied force by the z-feedback control and maintained constant contact of the cantilever with the sample surface. A comparison between DPRM and conventional PFM is shown in Fig. 5(b). In conventional PFM, a small converse piezoelectric response of approximately 100 pm must be detected. Because of the existence of electrostatic or electrochemical effects, the accuracy of the piezoelectric response has been noted. On the other hand, DPRM uses the direct piezoelectric response, which is hardly influenced by the other effect due to no voltage application. Moreover, the application of force is accurately controllable using a piezoelectric actuator. Given the calculated results shown in Fig. 1 and the experimental results shown in this paper, it is suggested that DPRM has the ability for quantitative analysis.

The piezoelectric response was detected by the output current using a lock-in-amplifier and *I*/*V* converter to prevent the formation of parasitic capacitance between the conductive cantilever beam and the bottom electrode of the sample (Fig. S5). Ideally, the output current (*I*_*p*_) is proportional to the frequency of the applied force because the charge induced via the direct piezoelectric effect is proportional to the force and the output current is given by the charge differentiation. Given that the cutoff frequency of the *I*/*V* converter was 10 kHz, all the measurements were carried out at 7.3 kHz.

In the measurements, a commercially available conductive Pt/Cr-coated AFM probe (Budget Sensors: ElectriTap190) with a radius of 25 nm and a spring constant of 48 N/m was attached to a film sample. The sample was vibrated along the longitudinal axis by a laminated piezoelectric actuator that was operated by an nf-function generator (WF1965). A periodic compressive strain was applied to the film at a frequency of 7.3 kHz. The charge induced via the direct piezoelectric response was measured using a lock-in amplifier (NF LI5640). Ferroelectric domain images were obtained by measuring the direct piezoelectric response while scanning a specific area under constant force modulation.

The current induced via the direct piezoelectric response is described as *I*_*P*_ sin(*ωt* + *θ*), where *I*_*P*_ is the amplitude of the induced current, *ω* is angular velocity, *t* is time and *θ* is phase. Therefore, the electric displacement component (*D*_3_) can be derived from

M1 $${D}_{3}={\int }_{0}^{t}\frac{{I}_{p}}{A}\,\sin (2\pi ft+\theta )dt=-\frac{{I}_{p}}{2\pi fA}\,\cos (2\pi ft+\theta ),$$

where *A* is the contact area of the tip. Using the piezoelectric constitutive equations

M2 $$\begin{array}{c}{D}_{i}={d}_{ij}{T}_{j}+{\varepsilon }_{ik}^{T}{E}_{k}={e}_{ij}{S}_{j}+{\varepsilon }_{ik}^{S}{E}_{k},\\ i,k=1,2,3\,j=1,2,\ldots ,6,\end{array}$$

where *S*_*ij*_ is the strain, ${\varepsilon }_{ik}^{S}$ is the electrical permittivity under constant strain, *T*_*j*_ is the stress, and *E*_*k*_ is electric field, *e*_33_, *f* can be written as

M3 $${e}_{33,f}=\frac{{I}_{p}}{2\pi fA{\varepsilon }_{33}}$$

The strain applied to the film was calculated by

M4 $${\varepsilon }_{33}=\frac{kx}{AE}$$

The equation used to calculate *e*_33_, *f* (Eq. (1)) was obtained from Eqs. (M.3) and (M.4).

The mapping measurement of PFM was carried out by applying an AC voltage of 1.0 V_rms_. The eight equivalent directions for the polarisation vector in the (100) epitaxial film were visualised by rotating the sample in −55° increments around the (010) plane and combining the out-of-plane and in-plane converse piezoelectric responses. The details are described in Fig. S3^32^.

## Supplementary information

Supplementary material

## Data Availability

The data that support the findings of this study are available upon request from the corresponding author.
