# Supplementary material for: Quantitative analysis of the direct piezoelectric response of bismuth ferrite films by scanning probe microscopy
Source: Sci Rep. 2019 Dec 23;9:19727. doi: 10.1038/s41598-019-56261-w (PMC6928215; doi:10.1038/s41598-019-56261-w)
Supplement: Supplementary file 1 — Supplementary material [file 41598_2019_56261_MOESM1_ESM.pdf]

Supplementary material

Quantitative analysis of the direct piezoelectric response of bismuth ferrite films by scanning probe microscopy

Kento Kariya, Takeshi Yoshimura*, Katsuya Ujimoto and Norifumi Fujimura

Department of Physics and Electronics, Graduate School of Engineering, Osaka Prefecture University, Sakai 599-8531, Japan

*Email: tyoshi@pe.osakafu-u.ac.jp

1 Dependence of DPRM mapping images on compressive force

DPRM measurements were carried out under compressive mechanical force modulation from 600 to 2300 nN. Figure S1(a)–(h) show the morphology and direct piezoelectric response of the (100) $BiFeO_3$ film measured at various forces. All the mapping images have a similar pattern. Although it appears that $e_{33,f}$ decreased when the applied force was more than 1300 nN, no change of the domain structure, including polarisation switching, was observed.

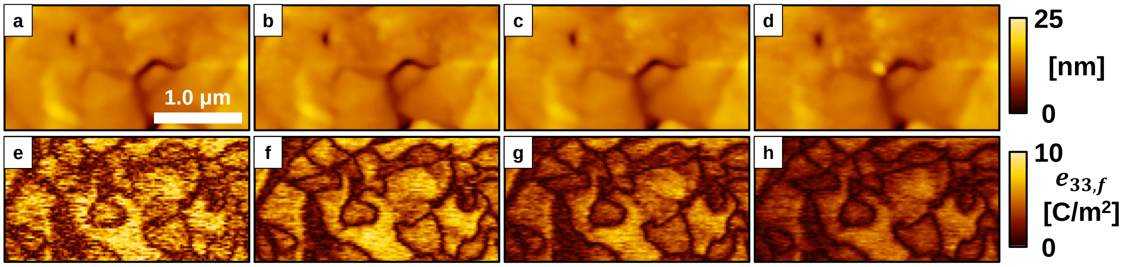

Figure S1. Change of the topography (top) and piezoelectric response (bottom) of a (100) $BiFeO_3$ epitaxial film with increasing mechanical compressive force modulation. Images obtained at forces of (a), (e) 600 nN, (b), (f) 1300 nN, (c), (g) 1600 nN, and (d), (h) 2300 nN.

2. Macroscopic $e_{31,f}$ versus nanoscopic $d_{33,AFM}$

The macroscopic $e_{31,f}$ and nanoscopic $d_{33,AFM}$ coefficients were characterised for oriented and epitaxial $BiFeO_3$ films[1–4] prepared by different methods under various conditions. The former coefficient was measured by bending the substrate and collecting the resultant charges, and the latter was determined from the strain curve obtained by piezoelectric force microscopy (PFM). The results are summarised in Fig. S2. No strong relationship between each piezoelectric response was observed.

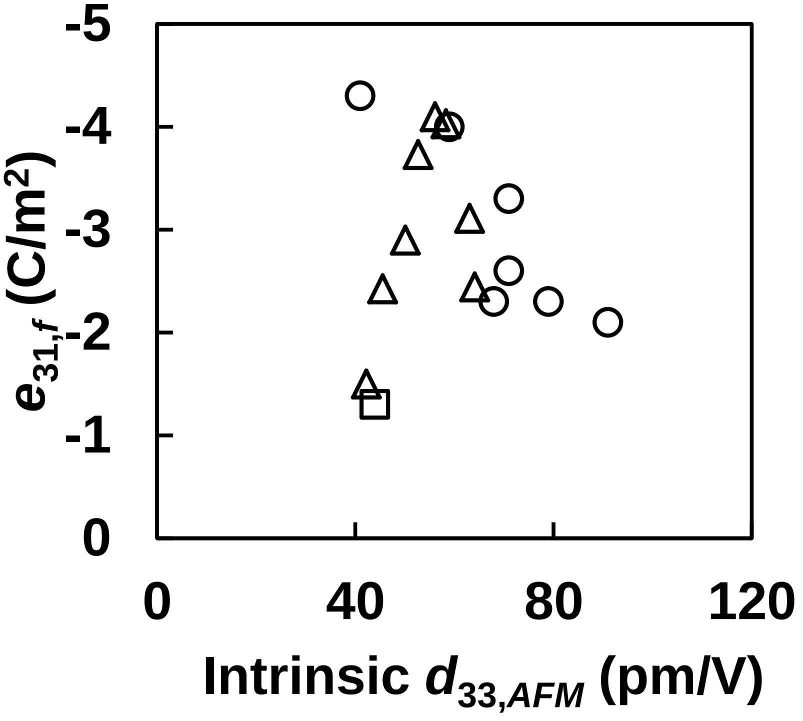

Figure S2. Dependence of the intrinsic coefficient on the macroscopic $e_{31,f}$ coefficient. Circles, squares, and triangles show the data for (100) epitaxial, (111) epitaxial, and (100) oriented $BiFeO_3$ films, respectively.

3. Determination of domain structure by PFM

PFM was used to determine the types (orientations) of domain walls[5]. To distinguish four variants of the in-plane direction of the spontaneous polarisation, the angle between the direction of the cantilever beam and <100> of $BiFeO_3$ was set at 55°. A schematic illustration of the measurement is shown in Fig. S3(a). The out-of-plane and in-plane directions of the spontaneous polarisation were determined by vertical and lateral PFM measurements, respectively, as shown in Supplementary Fig. S3(b) and (c), respectively. Figure S3(d) and (e) show the results of vertical and lateral PFM measurements for the (100) $BiFeO_3$ epitaxial film, respectively. From these results, the directions of the spontaneous polarisation for each domain were determined, as depicted in Fig. S3(f). From the relationship between the directions of the spontaneous polarisation in the adjacent domains, the types of domain walls, i.e. those with orientations of 71°, 109°, and 180°, were determined, as shown in Fig. 4(b) in the main text.

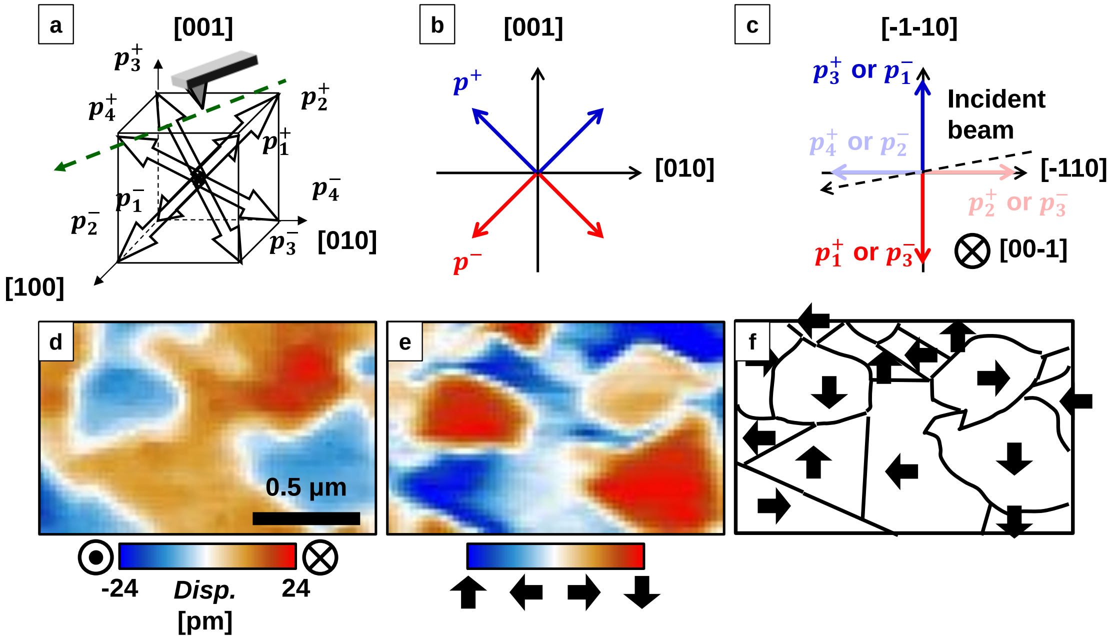

Figure S3. Domain wall detection by PFM. (a) Schematic of dipole vectors in a unit cell. The relationship between detected signals and depicted colour in (b) vertical and (c) lateral converse piezoelectric responses. (d) Vertical and (e) lateral domain pattern images of a (100) $BiFeO_3$ epitaxial film obtained by PFM measurement. (f) The visualised lateral domain vector image of the film.

4 Other results of DPRM mapping and the domain structure of (100) $BiFeO_3$ films

Figure S4 shows the DPRM mapping and the domain structure of the (100) $BiFeO_3$ film in areas other than domain C. The enhancement of the $e_{33,f}$ coefficient determined by DPRM in the domains with 71° domain walls was reproducibly observed.

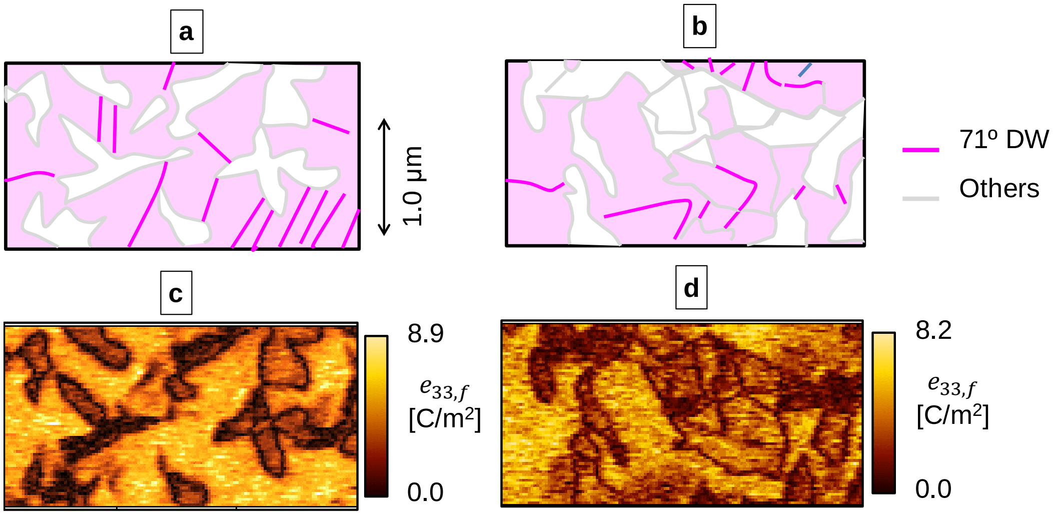

Figure S4. Results of DPRM mapping and the domain structure of (100) $BiFeO_3$ films in areas other than domain C. (a), (b) The domain structure determined by PFM. Purple lines indicate the 71° domain walls (DW). Light purple regions indicate the domains defined by the 71° domain walls. (c), (d) the corresponding DPRM images, respectively.

5 Detection of piezoelectric current by an *I*/*V* converter

When a conductive cantilever is attached to a film, a parasitic capacitance of more than 10^{-12} F, which is much higher than the sample capacitance (below 10^{-19} F in the case of a $BiFeO_3$ film), is formed. To avoid the effect of this parasitic capacitance, an *I*/*V* converter was used. The equivalent circuit of this measurement is shown in Supplementary Fig. S5(a). R_i and C_i of the *I*/*V* converter were 1.0 MΩ and 3.3 pF, respectively. The calculated frequency characteristics of the *I*/*V* converter are shown in Supplementary Fig. S5(b). The gain was constant up to 10 kHz. Therefore, the DPRM measurement was performed at 7.3 kHz.

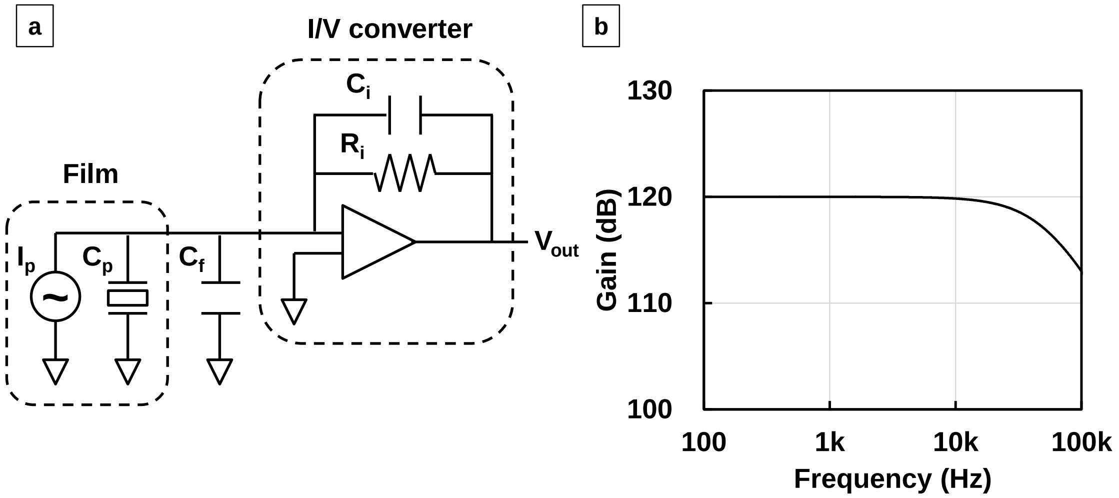

Figure S5. Piezoelectric current detection by the *I*/*V* converter. (a) Equivalent circuit when a cantilever is attached to the surface of the sample. The induced piezoelectric current is detected by the *I*/*V* converter. (b) Dependence of frequency on gain.

References

1. Kawahara, Y.; Ujimoto, K.; Yoshimura T.; Fujimura, N. Control of Crystal Structure of $BiFeO_3$ Epitaxial Thin Films by the Growth Condition and the Piezoelectric Properties. *Jpn. J. Appl. Phys.* **2012**, 51, 09LB04.

2. Yoshimura, T.; Ujimoto, K.; Kawahara, Y.; Wakazono, K.; Kariya, K.; Fujimura, N.; Murakami, S. Enhancement of direct piezoelectric properties of domain-engineered (100) $BiFeO_3$ films. *Jpn. J. Appl. Phys.* **2013**, 52, 09KA03.

3. Kariya, K.; Yoshimura, T.; Murakami, S.; Fujimura, N. Piezoelectric properties of (100) orientated $BiFeO_3$ thin films on $LaNiO_3$. *Jpn. J. Appl. Phys.*, **2014**, 53, 08NB02

4. Kariya, K.; Yoshimura, T.; Murakami, S.; Fujimura, N. Enhancement of piezoelectric properties of (100)-orientated $BiFeO_3$ films on (100)$LaNiO_3$/Si *Jpn. J. Appl. Phys.* **2014**, 53, 09PA14

5. Kim, B.; Barrows, F. P.; Sharma, Y.; Katiyar, R. S.; Phatak, C.; Amanda, K.; Petford-Long, Jeon, S., Hong, S. Ferroelectric Domain Studies of Patterned (001) $BiFeO_3$ by Angle-Resolved Piezoresponse Force Microscopy. *Sci. Rep.* **2018**, *8*, 23.
